# Cumulative lifetime acute stressor exposure interacts with reward responsiveness to predict longitudinal increases in depression severity in adolescence

**DOI:** 10.1017/S0033291722001386

**Published:** 2022-06-09

**Authors:** Kreshnik Burani, C. J. Brush, Grant S. Shields, Daniel N. Klein, Brady Nelson, George M. Slavich, Greg Hajcak

**Affiliations:** 1 Department of Psychology, Florida State University, Tallahassee, FL, USA;; 2 Department of Psychological Science, University of Arkansas, Fayetteville, AR, USA;; 3 Department of Psychology, Stony Brook University, Stony Brook, NY, USA; 4 Department of Psychiatry and Biobehavioral Sciences, University of California, Los Angeles, CA, USA

**Keywords:** Adolescent, Developmental Psychopathology, Life Stress, Major Depressive Disorder, Reward, RewP

## Abstract

**Background.:**

Life stress and blunted reward processing each have been associated with the onset and maintenance of major depressive disorder. However, much of this work has been cross-sectional, conducted in separate lines of inquiry, and focused on recent life stressor exposure, despite the fact that theories of depression posit that stressors can have cumulative effects over the lifespan. To address these limitations, we investigated whether acute and chronic stressors occurring over the lifespan interacted with blunted reward processing to predict increases in depression over time in healthy youth.

**Method.:**

Participants were 245 adolescent girls aged 8–14 years old (*M*_age_ = 12.4, s.d. = 1.8) who were evaluated at baseline and two years later. The reward positivity (RewP), an event-related potential measure of reward responsiveness, was assessed at baseline using the doors task. Cumulative lifetime exposure to acute and chronic stressors was assessed two years later using the Stress and Adversity Inventory for Adolescents (Adolescent STRAIN). Finally, depressive symptoms were assessed at both baseline and follow-up using the Children’s Depression Inventory.

**Results.:**

As hypothesized, greater lifetime acute stressor exposure predicted increases in depressive symptoms over two years, but only for youth exhibiting a blunted RewP. This interaction, however, was not found for chronic stressors.

**Conclusions.:**

Lifetime acute stressor exposure may be particularly depressogenic for youth exhibiting a blunted RewP. Conversely, a robust RewP may be protective in the presence of greater acute lifetime stressor exposure.

Major depressive disorder (MDD) is a pervasive and recurrent psychiatric condition that causes a substantial disease burden in adolescents and adults (Slavich & Irwin, 2014). In the United States, the lifetime prevalence is approximately 19%, with similar estimates documented in other countries (Bromet et al., 2011). Individuals with MDD often report considerable social and occupational impairment (Kessler et al., 2008; Kupferberg, Bicks, & Hasler, 2016). The economic burden of depression is also high. For example, in 2010, adults with MDD incurred approximately $100 billion in direct costs related to medical and pharmaceutical services sought for MDD, and another $100 billion in work-related costs attributable to days missed at work and lack of productivity (Greenberg, Fournier, Sisitsky, Pike, & Kessler, 2015). Among those who have experienced one lifetime major depressive episode (MDE), 60% are likely to develop a second MDE, with recurrence rates increasing as an individual experiences more MDEs (Monroe & Harkness, 2011; Solomon et al., 2000). MDD is thus a relatively common and highly burdensome disorder.

Major life stressors are one of the strongest known precipitants of MDD (Hammen, 2005; Kessler, 1997; Slavich, 2016), with their occurrence substantially increasing the risk for developing depressive symptoms and MDD in both adolescents and adults (Bouma, Ormel, Verhulst, & Oldehinkel, 2008; Fried, Nesse, Guille, & Sen, 2015; Ge, Lorenz, Conger, Elder, & Simons, 1994; Kendler, Karkowski, & Prescott, 1999; Mazure, 1998; van Praag, 2004). Stressor duration also has been found to be an important dimension associated with risk for depressive symptoms and MDD (Carter & Garber, 2011; Slavich, Stewart, Esposito, Shields, & Auerbach, 2019). Both acute and chronic stressors have been related to the onset of a depressive episode (Hammen, Kim, Eberhart, & Brennan, 2009); however, research suggests that acute stressors may be particularly depressogenic (Monroe, Slavich, & Georgiades, 2009; Slavich, O’Donovan, Epel, & Kemeny, 2010).

Despite the abundance of research examining the association between life stressor exposure and depression, much of this work has focused on recent life stressors. In addition, the neurobiological mechanisms linking stress with depression remain unclear. One possible pathophysiological mechanism involves stress-induced reductions in reward-related brain activity that may lead to increases in depression (Pizzagalli, 2014). For example, experimental research using functional magnetic resonance imaging (fMRI) provides support indicating that laboratory stressors blunt activity in the mesolimbic and mesocortical reward pathways (Kumar et al., 2014; Lincoln et al., 2019; Porcelli, Lewis, & Delgado, 2012). Moreover, electroencephalogram (EEG) research has identified the reward positivity (RewP) as an index of reward processing evident approximately 250 ms to 350 ms following feedback indicating rewards compared to losses (Proudfit, 2015). Prior research has shown that the RewP has good internal consistency in both adolescents and adults (Levinson, Speed, Infantolino, & Hajcak, 2017; Luking, Nelson, Infantolino, Sauder, & Hajcak, 2017) and that it relates to other measures of reward processing. Specifically, a larger RewP has been associated with greater self-reported reward responsiveness, ventral striatum activation, and reward response bias (Becker, Nitsch, Miltner, & Straube, 2014; Bress & Hajcak, 2013; Carlson, Foti, Mujica-Parodi, Harmon-Jones, & Hajcak, 2011). Overall, the RewP has good psychometric properties and construct validity.

A reduced RewP has been shown to be evident among children and adults with increased depressive symptoms and MDD (Belden et al., 2016; Bowyer et al., 2019; Bress, Smith, Foti, Klein, & Hajcak, 2012; Brush, Ehmann, Hajcak, Selby, & Alderman, 2018; Foti & Hajcak, 2009; Klawohn, Burani, Bruchnak, Santopetro, & Hajcak, 2020; Liu et al., 2014). Moreover, a reduced RewP has been observed prior to the onset of depression, suggesting that it may be a pre-clinical marker of subsequent risk for MDD (Bress, Foti, Kotov, Klein, & Hajcak, 2013; Nelson, Perlman, Klein, Kotov, & Hajcak, 2016). Collectively, the evidence indicates that the RewP is a reliable neural indicator of reward processing that is frequently blunted in individuals with elevated depressive symptoms and MDD and reduced among those at increased risk for depression (Proudfit, 2015).

In addition, prior research has indicated that reward processing, as measured by the RewP, interacts with life stressor exposure to predict increases in depressive symptoms in adolescents and adults. In a cross-sectional study of young adults, Pegg et al. (2019) examined whether social *v*. non-social lifetime stressor severity as measured by the Stress and Adversity Inventory for Adults (STRAIN) interacted with the RewP, measured during a social reward task, to predict depressive symptoms. The authors found that greater lifetime social stressor severity interacted with a blunted RewP to social rewards to predict greater increases in depressive symptoms; however, this was not found for non-social stress severity. In a separate longitudinal study using the Adolescent Life Events Questionnaire, Burani et al. (2021) found that experiencing more recent stressful life events interacted with a blunted RewP to predict greater increases in depressive symptoms one year later in adolescent girls. Similarly, Goldstein et al. (2020) found that experiencing more stressful life events, as measured using the UCLA Life Stress Interview, interacted with a blunted RewP to predict greater increases in depressive symptoms three years later. Collectively, these cross-sectional and longitudinal studies are consistent with a diathesis-stress model of depression (Monroe & Simons, 1991), wherein life stressors are most likely to precipitate depression for persons exhibiting a blunted RewP. To our knowledge, however, no studies have examined how stressors occurring over the entire life course interact with this neural marker indicator of reward-related brain activity to predict changes in depressive symptoms in adolescents over time.

The absence of lifetime stressor studies on this topic is notable because cumulative lifetime stressor exposure has been hypothesized to have adverse effects on neurobiological systems such as the hypothalamic–pituitary–adrenal (HPA) axis (Lupien, McEwen, Gunnar, & Heim, 2009), the reward system (Casement, Shaw, Sitnick, Musselman, & Forbes, 2015), and the immune system (Slavich, 2020; Slavich & Irwin, 2014), each of which have been associated with depression. Stressor exposure activates the HPA axis, which leads to the production of cortisol that helps to mobilize the body’s response to stressors; however, repeated exposure to stressors can cause prolonged hyperactivation of the HPA axis that has health-damaging effects (Lupien et al., 2009). Similarly, repeated exposure to stressors during adolescence predicts blunted activation of the ventral striatum – a brain region linked to the RewP – during adulthood (Hanson et al., 2016). Likewise, chronic and repeated stressor exposure has been found to contribute to increased inflammatory activity (Furman et al., 2019), which has been implicated in depression (Slavich & Irwin, 2014). Therefore, multiple findings underscore the importance of investigating how cumulative lifetime stressor exposure interacts with the RewP to shape the risk for stress-related disorders such as depression.

The present study addresses this issue by examining whether acute and chronic stressors occurring over the life course prospectively predict increases in depressive symptoms in the context of a blunted RewP in a large community sample of 8-to-14-year-old girls who were assessed longitudinally over two years. Acute stressors were defined as those lasting a few days (e.g. hearing bad news, getting into an accident), whereas chronic stressors were defined as those lasting at least one month (e.g. persistent housing, financial, or relationship problems; Slavich et al., 2019). Recent evidence suggests that acute stressors are stronger proximal predictors of MDD as compared to chronic stressors (Monroe et al., 2009; Slavich et al., 2010). Therefore, we were particularly interested in whether acute stressor exposure interacts with the RewP to predict subsequent increases in depressive symptoms.

To investigate this possibility, at baseline, our sample of adolescent females completed a reward task while EEG data were collected; they also completed the Children’s Depression Inventory (CDI) to assess their depressive symptoms. Two years later, these participants again completed the CDI as well as the Adolescent STRAIN to assess their lifetime stressor exposure. Based on the literature summarized above, we hypothesized that the RewP assessed at baseline would interact with participants’ lifetime stressor exposure to predict subsequent increases in depression. Given prior research on the topic (e.g. Monroe et al., 2009; Slavich et al., 2010), we expected that acute lifetime stress exposure, in particular, would be most strongly related to increases in depression for youth exhibiting a blunted RewP.

## Method

### Sample

Participants were recruited from Long Island, New York, as part of a longitudinal study examining processes associated with developmental changes in reward processing. The complete sample included 317 adolescent girls between 8 and 14 years old (*M*_age_ = 12.4, s.d. = 1.8). Of these 317 participants, three did not complete the reward task and nine were excluded due to poor EEG data quality. Therefore, the final sample at baseline included 305 participants (*M*_age_ = 12.4, s.d. = 1.8). At baseline, participants completed the reward task and the CDI (see below).

Two years later, participants returned for a follow-up visit, during which time STRAIN and CDI data were collected (*n* = 245; *M*_age_ = 14.4, s.d. = 1.8). Participants who completed both the baseline visit and follow-up visit were younger than those who only completed the baseline visit (*M*_diff_ = 0.52, *t*(303) = 2.06, *p* = 0.040). Importantly, these two groups did not differ on baseline CDI or RewP scores (*ps* = 0.667).

At the follow-up visit, participants were predominantly White (87%) with the remaining participants self-identifying as African American (6.5%), American Indian/Alaskan Native (0.8%), or Other (5%). Nine percent of the sample at the follow-up visit reported being Hispanic. All participants also completed the Kiddie Schedule for Affective Disorders and Schizophrenia (KSADS) at baseline. Of the 245 participants included in the final sample, 6 individuals (~2.4% of the sample) met the diagnostic criteria for MDD according to the Diagnostic Statistical Manual of Mental Disorders-IV. The average household income of the final sample was $127 190 (s.d. = $75 159). All participants and their parents provided informed assent and consent, respectively, as approved by the Institutional Review Board at Stony Brook University.

### Stress and Adversity Inventory for Adolescent STRAIN

Lifetime stressor exposure was assessed at the follow-up session (*n* = 245) using the Adolescent STRAIN (Slavich et al., 2019; see https://www.strainsetup.com). Participants reported on 33 acute life events and 42 chronic difficulties for a total of 75 stressors that spanned 12 primary life domains (i.e. Housing, Education, Work, Treatment/Health, Marital/Partner, Reproduction, Financial, Legal/Crime, Other Relationships, Guardian/Parent Relationships, Death, Life-Threatening Situations) and five core social-psychological characteristics (i.e. Interpersonal Loss, Physical Danger, Humiliation, Entrapment, Role Change/Disruption). For each stressor endorsed, the STRAIN generates additional questions assessing the stressor’s severity, frequency, timing of exposure, and duration.

Acute life events (i.e. acute stressors) in the STRAIN system are defined as stressors typically lasting a few days, such as learning of a death, getting fired, or being physically attacked (Slavich et al., 2019). Chronic difficulties (i.e. chronic stressors), in turn, typically last a minimum of one month though many last longer, such as persistent educational, housing, or financial problems (Slavich et al., 2019). Based on participants’ responses, the STRAIN produces cumulative lifetime stressor indices summarizing each individual’s total lifetime stressor exposure (i.e. number of stressors experienced) and severity, the latter of which is based on a five-point Likert stressor severity scale. These scores can then be calculated separately for acute and chronic stressors. The STRAIN assesses a variety of stressors across 12 life domains. Higher scores always represent greater lifetime stressor exposure. The Adolescent STRAIN has been validated against clinical, cognitive, and behavioral outcomes (Slavich et al., 2019) as has its sister instrument, the Adult STRAIN (Banica, Sandre, Shields, Slavich, & Weinberg, 2020, 2021; Cazassa, da Oliveira, Spahr, Shields, & Slavich, 2020; Slavich & Shields, 2018; Sturmbauer, Shields, Hetzel, Rohleder, & Slavich, 2019).

### Reward task

The doors task is a simple monetary reward paradigm in which participants are presented with two doors displayed side-by-side and are instructed to select the door they believe will yield a prize (i.e. money) using the left or right mouse button. Once participants make a decision, a fixation cross is presented for 1500 ms followed by feedback indicating whether they won (i.e. green arrow pointing upwards signifies +$0.50) or lost (i.e. red arrow pointing downwards signifies −$0.25); this feedback was presented for 2000 ms. Following each trial, text on the screen instructed participants to ‘Click for next round,’ followed by a fixation cross presented for 1000 ms. There was a total of 30 gain and 30 loss trials, presented pseudorandomly using Presentation version 17.0 (Neurobehavioral Systems, Albany, CA). Participants were told they had a chance to earn up to $15; all participants were given $8 at the end of the task.

### Children’s Depression Inventory (CDI)

The CDI assesses depressive symptoms over the last two weeks and has been well-validated in children between 7 and 17 years old (Kovacs, 1992). It consists of 27 items rated on a 3-point Likert scale ranging from 0 to 2, with 0 indicating an absence of a symptom and 2 indicating the definite presence of the symptom. Total scores range from 0 to 54, with higher scores indicating greater depressive symptom severity. Participants completed the CDI at baseline (*n* = 305; *α* = 0.89) and two years later (*n* = 255; *α* = 0.90).

### EEG processing

Continuous EEG data were recorded while participants completed the doors task using the ActiveTwo BioSemi system (BioSemi, Amsterdam, Netherlands) using an elastic cap containing 34 electrode sites placed according to the 10/20 system (i.e. 32 channels plus Iz and FCz). Facial electrodes were placed above and below the left eye, and near the outer canthi of the left and right eyes to monitor horizontal and vertical electrooculographic activity. Two additional electrodes were placed on the left and right mastoids. The EEG signal was pre-amplified at the electrode to improve signal-to-noise ratio, and data were digitized at a 24-bit resolution with a sampling rate of 1024 Hz using a low-pass fifth-order sinc filter with a half-power cutoff of 204 Hz. Active electrodes were measured online with reference to a common mode sense active electrode constructing a monopolar channel.

EEG data were processed offline using BrainVision Analyzer 2.1 (Brain Products, Gilching, Germany). Raw EEG data were re-referenced to the average of the left and right mastoids. The re-referenced data were filtered from 0.1 Hz to 30 Hz using a 2nd order filter. The EEG data were then segmented from −200 ms prior to the onset of feedback and up to 1000 ms following feedback. Eyeblinks and ocular movement correction was performed using the Gratton, Coles, and Donchin (1983) regression-based method. Prior to averaging the data as a function of feedback type, segments containing a voltage greater than 50 μV between consecutive sample points, a voltage difference of 300 μV within a segment, or a maximum voltage difference of less than 0.5 μV within 100-ms intervals were identified as artifacts and automatically rejected. The 200-ms pre-feedback interval was used for baseline correction.

### RewP quantification

The RewP was calculated as the mean activity within a 100-ms time window around the most positive peak of the gain (*M*_trials_ = 29.3; s.d. = 1.8) minus loss (*M*_trials_ = 29.2; s.d. = 1.9) difference waveform extracted from a 200 ms to 400 ms time window at FCz for each participant (Burani et al., 2021). No participants were excluded for having a low number of trials.

### Data analysis

All analyses were conducted in R version 4.0.2 (R Core Team, 2020). First, bivariate Pearson correlations were conducted to determine associations between the Adolescent STRAIN variables, Baseline RewP, Baseline Age, and CDI scores at baseline and follow up using the *psych* R package (Revelle, 2020). Internal consistency was calculated using the *Psychometric* package (Fletcher, 2010). To test our primary hypothesis, a multiple linear regression was conducted. CDI scores at follow up were predicted from Baseline RewP, Baseline Age, Baseline CDI scores, Lifetime Acute Stressor Exposure, Lifetime Chronic Stressor Exposure, the Lifetime Acute Stressor Exposure × Baseline RewP interaction, and the Lifetime Chronic Stressor Exposure × Baseline RewP interaction. All variables were mean-centered in the regression analyses. For the multiple linear regression, we used the stats R package (R Core Team, 2020), and follow-up simple slopes analyses were performed and plotted using the *interactions* package (Long, 2019). The Johnson-Neyman technique was used to identify the region of significance wherein the simple slope analyses showed a significant interaction (Johnson & Neyman, 1936). The Johnson-Neyman plot thus provides values of the moderator for which the relation between the predictor and outcome are both non-significant and significant. The final sample size for the regression analyses was 245.

To test for the robustness of any significant effect observed, we also performed sensitivity analyses that included household income and ethnicity as additional covariates. The household income variable was recoded into a categorical variable prior to being entered into the regression such that an increase of $ 30 000 placed participants into a higher category (e.g. 0 = less than $29900, 1 = between 29 900 and 59 000, etc., up to 299 900). All participants who reported an income of more than $ 299 900 were all placed in one category. Given that the sample consisted largely of White participants, ethnicity was dichotomized into White (0) and non-White (1). The *car* R package was used to generate the variance inflation factor (Fox, 2020) which indicates the degree of multicollinearity.

## Results

### Descriptive statistics

**Fig. 1.**
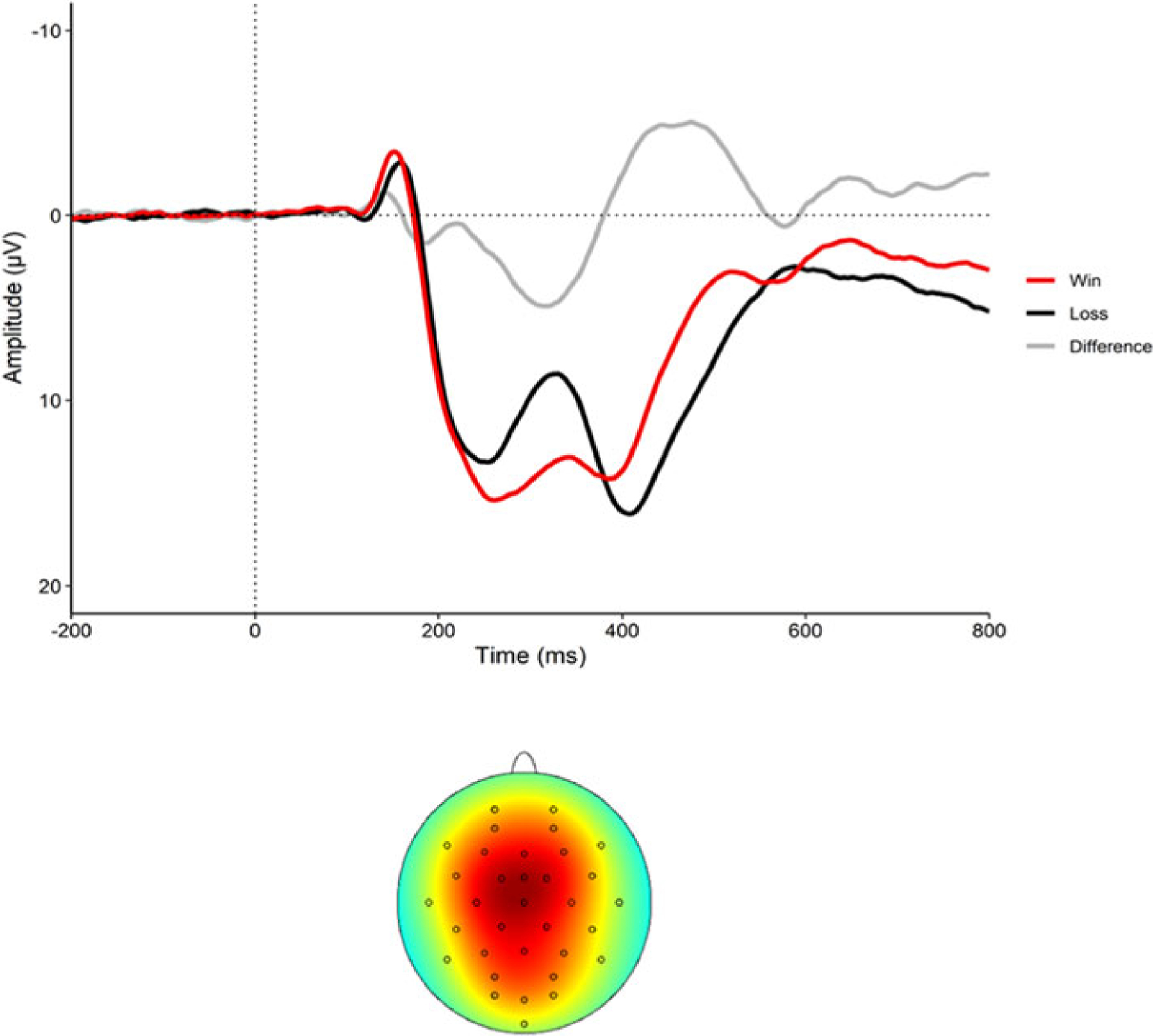
The event-related potential (ERP) to wins (red) and losses (black) as well as the difference waveform (RewP) at electrode FCz (top graph) and the scalp topography (bottom graph) (*N* = 245).

Figure 1 displays the event-related potential to wins and losses as well as the RewP (i.e. difference waveform) and the scalp topography of the sample. Demographics for the sample, and descriptives of all study variables, are presented in Table 1. Correlations between the variables are presented in Table 2. Participants experienced an average of 13.9 lifetime stressors (s.d.. = 10.63; range 0–56), with 8.1 acute stressors (s.d. = 6.52; range 0–34) and 5.8 chronic stressors (s.d. = 4.90; range 0–28). Average RewP amplitude at baseline was 5.53 μV (s.d. = 6.2). Of note, the RewP did not correlate with any lifetime stressor exposure variables (see Table 2).

### Lifetime chronic and acute stressor exposure

**Fig. 2.**
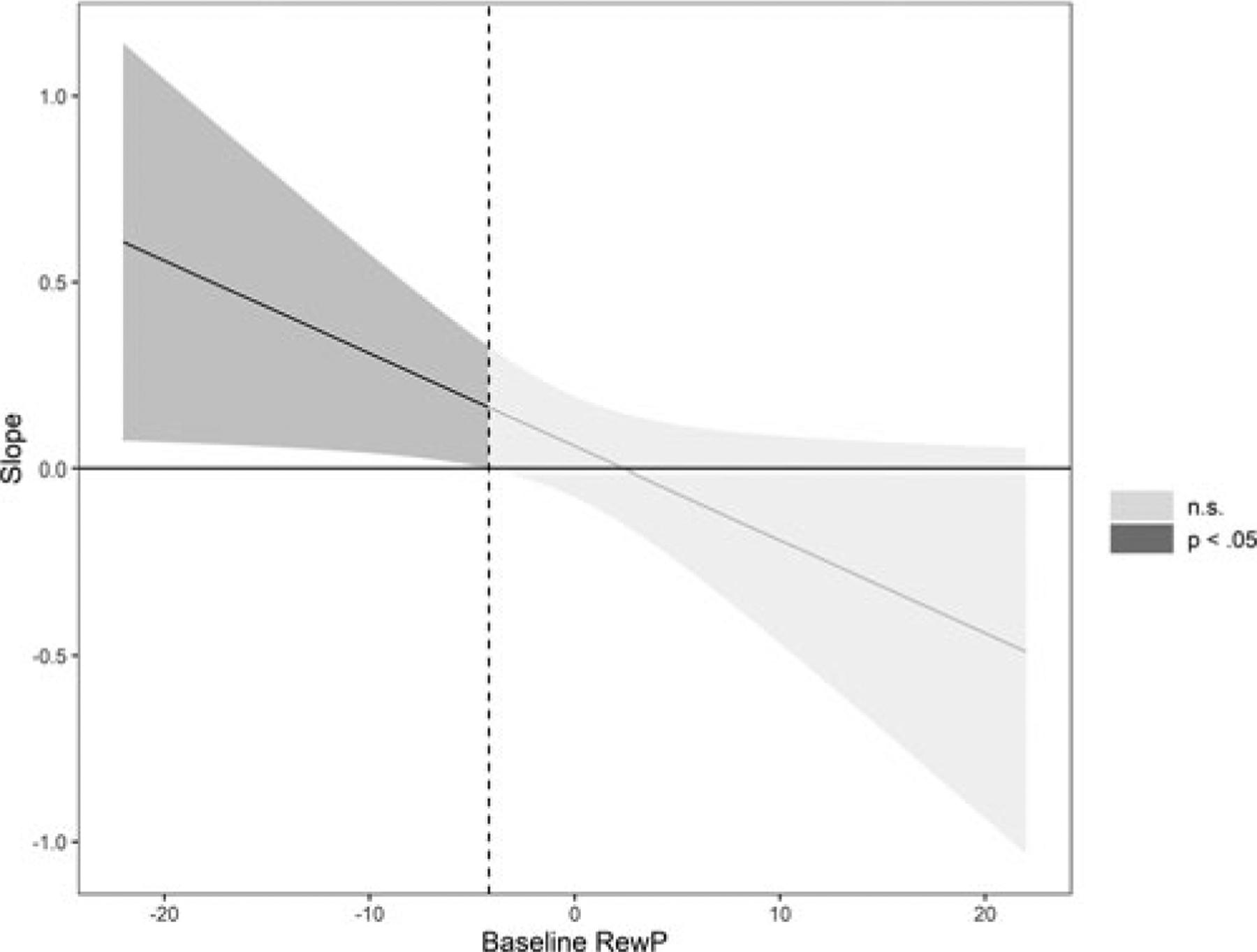
Conditional effect of lifetime acute stressor exposure (STRAIN) on follow-up depressive severity (CDI). Johnson-Neyman plot of the Lifetime Acute Stressor Exposure × Reward Positivity interaction predicting depressive symptom scores on the Children’s Depression Inventory at follow up. The dotted line indicates the value of the RewP where the confidence intervals cross zero. Lifetime Acute Stressor Exposure predicted depressive symptom scores at follow up at amplitudes of RewP below the dotted line. The shaded area represents the confidence interval of the conditional effect, with the dark gray area representing values of Baseline RewP for which the effect is significant, and the light gray area representing values of Baseline RewP for which the effect is non-significant. Baseline RewP, Age, CDI scores and Lifetime Chronic Stressor Exposure were included as covariates (*N* = 245).

We examined whether lifetime acute stressor exposure and lifetime chronic stressor exposure interacted with baseline RewP to predict depressive symptoms at follow up, controlling for baseline depressive symptoms and baseline age (Table 3). Higher baseline depressive symptoms and greater lifetime chronic stressor exposure predicted higher depressive symptoms at follow up. Baseline RewP, baseline age and lifetime acute stressor exposure, however, did not predict elevations in depressive symptoms at follow up. Finally, lifetime acute, but not chronic, stressor exposure interacted with baseline RewP to predict greater depressive symptoms at follow up. Simple slopes analyses indicated that lifetime acute stressor exposure predicted higher depressive symptoms at follow up for youth with a low (−1 s.d.) but not average or high (+1 s.d.) RewP (see Table 4 and Fig. 2).

Online Supplementary Table S1 contains a multiple regression model predicting depressive symptoms at follow up with the same predictors as the main regression model but, in addition, includes household income and ethnicity as covariates. With household income and ethnicity included, lifetime acute stressor exposure and the RewP interacted to predict depressive symptoms at follow up at a trend level. These results suggest that income and ethnicity may partially explain this interactive effect on depression.

### Lifetime chronic and acute stressor severity

Online Supplementary Table S2 contains the results of a multiple regression model predicting CDI scores at follow up from the lifetime acute stressor severity and lifetime chronic stressor severity variable while controlling for baseline RewP, baseline CDI and baseline age. This analysis was conducted to examine the specificity of effects for stressor exposure v. stressor severity. Baseline age, baseline RewP, and lifetime acute stressor severity were not significant predictors of depression at follow up; however, greater baseline depressive symptoms and lifetime chronic stressor severity predicted higher follow-up depressive symptoms. Finally, lifetime acute, but not chronic, stressor severity interacted with the RewP to predict increases in depressive symptoms at follow up such that lifetime acute stressor severity predicted depressive symptoms but only for those with a blunted RewP (see online Supplementary Table S2a). Therefore, the results for lifetime acute stressor exposure and severity were similar.

### Residualized wins and residualized losses

To examine whether these results were driven by wins or losses, we used residualized difference scores for both wins and losses (Meyer, Lerner, De Los Reyes, Laird, & Hajcak, 2017) in separate regression models. Overall, the results indicated that only residualized wins interacted with lifetime acute stressor exposure to predict depressive symptoms at follow up. In sum, therefore, these data suggest that the results may be driven by neural responses to rewards instead of losses (see online Supplementary Tables S3, S3a and S4).

## Discussion

The present study examined whether stressors occurring across the life course moderated the association between the RewP and longitudinal increases in depressive symptoms in a large sample of 8-to-14-year-old girls. We found that exposure to chronic (but not acute) stressors over the lifespan predicted increases in depressive symptoms over time. As hypothesized, however, only acute lifetime stressor exposure was moderated by the RewP, such that increases in depressive symptoms over the two-year study period were evident only for youth exhibiting a blunted RewP.

These results are consistent with diathesis-stress models of depression (Monroe & Simons, 1991) insofar as acute life stressors appeared to be particularly depressogenic for youth with blunted reward processing. These findings are also consistent with prior studies, which have found that life stress and the RewP interact to predict depression (Burani et al., 2021; Goldstein et al., 2020). Our results add to this prior work by suggesting that this interaction between life stressor exposure and reward functioning may be evident only for acute stressors occurring over the life course. The RewP may thus be a stress vulnerability marker insofar as individuals with a blunted RewP exhibit less responsivity to reward and reward-seeking behavior (Bress & Hajcak, 2013), which may lead to a greater impact of acute stressor exposure through less engagement with rewarding activities and stimuli in the environment.

One possible biological pathway by which stressor exposure may impact reward processing leading to depression involves the HPA axis. When activated, the HPA axis produces corticotropin-releasing factor (CRF) and glucocorticoids. Several brain regions implicated in reward processing are rich in receptors for CRF and glucocorticoids, including the prefrontal cortex, the ventral striatum, and the ventral tegmental area (Novick et al., 2018; Van Pett et al., 2000). Increased CRF in the reward system may in turn attenuate dopamine transmission leading to reduced reward-related behavior (Bryce & Floresco, 2016). A second possibility involves the immune system. Greater lifetime stressor exposure has been found to predict higher levels of inflammatory activity (Byrne et al., 2021), and a core biobehavioral effect of inflammation involves the reduction of reward-related neural signaling and behavior (Felger et al., 2016; Slavich, 2020, 2022). Additional research is needed to investigate these and other biological pathways that may link greater lifetime stressor exposure with reward-related neural function and depression.

Consistent with prior research, these results also demonstrate that a robust RewP may potentially confer resilience to stress (Burani et al., 2021; Goldstein et al., 2020; Pegg et al., 2019). Individuals with greater acute lifetime stressor exposure did not exhibit increases in depressive symptoms two years later if they had a larger RewP response to reward. This finding suggests that youth who respond to rewarding stimuli robustly may be relatively protected against the depressogenic effects of life stress. Importantly, hypersensitivity to rewards may also function as a risk factor for other forms of psychopathology. Increased reward processing, for example, could lead to excessive approach-related affect that is characteristic of hypomania and mania (Alloy & Nusslock, 2019; Nusslock, Young, & Damme, 2014). Nevertheless, future research may benefit from developing interventions that aim to enhance reactivity to pleasant stimuli/rewards among those with a blunted RewP, which may increase resiliency – something that is currently done in cognitive behavior therapy (e.g. promoting behavioral activation, scheduling pleasurable activities) but which could be perhaps further emphasized for those with deficient reward system functioning.

Several limitations of this study are worth noting. First, we only sampled adolescent girls given their relatively high risk for developing depression during this developmental period. Moreover, this sample was largely White. Future research should thus examine if these results generalize across both sexes and genders as well as to older adolescents, adults, and racially and ethnically diverse populations. This is particularly important as the nature of depressive symptoms differs across ethnicities (Assari & Moazen-Zadeh, 2016; Wight, Aneshensel, Botticello, & Sepúlveda, 2005) as well in adolescents v. adults, who display higher levels of anhedonia (Rice et al., 2019). Therefore, the age range and racial/ethnic composition of this sample may limit the depressive symptoms that were assessed. Along these same lines, additional research is needed to examine the possibility that any sex and/or gender differences detected in the future may vary as a function of biological and/or social factors that have been associated with depression. Second, participants were recruited from the community and thus exhibited relatively few major life stressors and depressive symptoms. It should be noted that even modest elevations in depressive symptoms in this age range are strong predictors of subsequent increases in depressive symptoms or onset of MDD (Klein, Shankman, Lewinsohn, & Seeley, 2009). Nevertheless, future studies are needed to study these processes in at-risk and depressed populations. Specifically, recruiting individuals who have experienced more lifetime stressors, or who are at risk for developing depression, would permit an examination of whether such stressors interact with blunted reward processing to predict caseness of MDD, rather than increases in depressive symptoms. In addition, future studies could investigate whether the timing of acute or chronic stressors interacts with the RewP to predict depressive symptoms.

Third, future studies are needed to examine the time course characteristics of both lifetime acute stressor exposure and blunted reward processing, and how they interact to impact the development of depressive symptoms. Fourth, we used baseline RewP as predictor of changes in depressive symptoms over time. This approach has both limitations and benefits. Regarding the limitations, although clinical interview data suggest that this sample was relatively healthy, it is unknown whether these adolescents experienced depressive symptoms prior to the baseline visit that may have impacted their RewP. Future longitudinal research is thus needed to examine this issue. Fifth, retro-spective self-reporting of life stressors can be biased by depressive symptoms, which can make it difficult to determine if high lifetime stressor scores are attributable to experiencing more stressors v. having more depressive symptoms (Harkness & Monroe, 2016). We addressed this issue by using an instrument for assessing stressor exposure (i.e. the STRAIN), which is not sensitive to negative mood or social desirability (Slavich & Shields, 2018).

Notwithstanding these points, the present study is the first to demonstrate that greater lifetime acute stressor exposure interacts with the RewP to predict longitudinal increases in depressive symptoms over a two-year period. Framed in terms of resilience, individuals with an increased RewP appeared to be protected from the depressogenic effects of acute life stressor exposure. In contrast with our prior work, we did not find a main effect of RewP in prospectively predicting increases in depressive symptoms (Nelson et al., 2016). However, the present study differed in several ways, including a different age range and different symptom measures (i.e. interview-based v. self-report), and a longer duration between baseline and follow up. In doing so, the present study extends our understanding of how the RewP interacts with lifetime stressor exposure to predict changes in depression over time. Collectively, these data provide novel evidence regarding possible pathways to depression, insofar as they suggest that adolescents who experience greater acute stressor burden over the lifespan, and who do not exhibit a strong neural response to reward, may be at the greatest risk of developing depression.

## Supplementary Material

Supplement

## Figures and Tables

**Table 1.** Descriptives for the study variables, and demographics of the sample (*N* = 245).

Variable	*M* (s.d.)	Min-Max	Skewness
Lifetime total stressor exposure (STRAIN)	13.9 (10.6)	0–56	1.21
Lifetime chronic stressor exposure (STRAIN)	5.8 (4.9)	0–28	1.06
Lifetime acute stressor exposure (STRAIN)	8.1 (6.5)	0–34	1.38
Baseline RewP (μV)	5.5 (6.2)	−16.70 to 21.37	−0.06
Baseline age	12.3 (1.8)	8.01–14.99	−0.52
Baseline depressive symptoms (CDI)	6.6 (6.4)	0–31	1.28
Follow-up depressive symptoms (CDI)	6.6 (6.5)	0–36	1.54
Ethnicity	N		
White	203		
African American	13		
Hispanic	11		
Other	11		
Income	*M* = $127 190 (s.d. = $75 159)		

STRAIN, Stress and Adversity Inventory; RewP, Reward Positivity; CDI, Children’s Depression Inventory.

*Note:* Of the 245 participants in the final sample, data on ethnicity were available for 238 participants.

**Table 2.** Correlations among the main study variables

	1.	2.	3.	4.	5.	6.	7.
Baseline age	1						
Baseline RewP	0.12*	1					
Baseline depressive symptoms (CDI)	0.24**	0.06	1				
Lifetime total stressor exposure (STRAIN)	0.29**	−0.01	0.48**	1			
Lifetime chronic stressor exposure (STRAIN)	0.30**	0.01	0.48**	0.91**	1		
Lifetime acute stressor exposure (STRAIN)	0.25**	−0.03	0.42**	0.95**	0.72**	1	
Follow-up depressive symptoms (CDI)	0.31**	0.02	0.60**	0.58**	0.27**	0.50**	1

STRAIN, Stress and Adversity Inventory; RewP, Reward Positivity; CDI, Children’s Depression Inventory.

** *p* < 0.001.

* *p* < 0.05.

Pearson’s *r* correlations are reported.

**Table 3.** Regression results using depressive symptoms at follow up as the dependent variable predicted from lifetime acute stressor exposure, lifetime chronic stressor exposure, baseline age, RewP, depressive symptoms, the lifetime acute stressor exposure × baseline RewP interaction, and the lifetime chronic stressor exposure × baseline RewP interaction

		*b*		
Predictor	*b*	95% CI [LL to UL]	*VIF*	Model fit
(Intercept)	6.59**	[6.00 to 7.19]	-	
Baseline RewP	−0.06	[−0.16 to 0.04]	1.06	
Baseline age	0.31^	[−0.05 to 0.66]	1.15	
Baseline depressive symptoms (CDI)	0.38**	[0.28 to 0.49]	1.36	
Lifetime acute stressor exposure (STRAIN)	0.06	[−0.07 to 0.19]	2.18	
Lifetime chronic stressor exposure (STRAIN)	0.44**	[0.26 to 0.62]	2.33	
Lifetime acute stressor exposure × baseline RewP	−0.02*	[−0.05 to −0.001]	2.14	
Lifetime chronic stressor exposure × baseline RewP	0.01	[−0.02 to 0.04]	2.13	
				*F*(7, 237) = 33.37**
				*R*^2^ = 0.482**
				95% CI [0.35, 0.53]

RewP, Reward Positivity; CDI, Children’s Depression Inventory; STRAIN, Stress and Adversity Inventory; b, unstandardized regression weights; CI, 95% confidence interval; LL and UL indicate the lower and upper limits of the CI, respectively; VIF, Variance Inflation Factor.

^ = *p* < 0.10;

* = *p* < 0.05;

** = *p* < 0.01.

**Table 4.** Simple slopes of the relation between lifetime acute stressor exposure (STRAIN) and depression severity (CDI) scores two years later at different levels of baseline RewP

Lifetime acute stressor exposure × RewP	*b*	Lower CI	Upper CI	*p*
RewP (−1 s.d.)	0.22	0.02	0.41	0.031***
RewP (*M*)	0.06	−0.07	0.19	0.372
RewP (+1 s.d.)	−0.10	−0.30	0.11	0.357

RewP, Reward Positivity; CI, 95% confidence interval.

*** *p* < 0.001.

*Note:* Beta coefficients are adjusted for baseline depressive symptoms and baseline age.

## References

[R1] Alloy LB, & Nusslock R (2019). Future directions for understanding adolescent bipolar spectrum disorders: A reward hypersensitivity perspective. Journal of Clinical Child & Adolescent Psychology, 48(4), 669–683. 10.1080/15374416.2019.1567347. 30908092 PMC6588455

[R2] Assari S, & Moazen-Zadeh E (2016). Ethnic variation in the cross-sectional association between domains of depressive symptoms and clinical depression. Frontiers in Psychiatry, 7, 53. 10.3389/fpsyt.2016.00053. 27148084 PMC4834296

[R3] Banica I, Sandre A, Shields GS, Slavich GM, & Weinberg A (2020). The error-related negativity (ERN) moderates the association between interpersonal stress and anxiety symptoms six months later. International Journal of Psychophysiology, 153, 27–36. 10.1016/j.ijpsycho.2020.03.006. 32277956 PMC7335004

[R4] Banica I, Sandre A, Shields GS, Slavich GM, & Weinberg A (2021). Associations between lifetime stress exposure and the error-related negativity (ERN) differ based on stressor characteristics and exposure timing in young adults. Cognitive, Affective, and Behavioral Neuroscience, 1–18. 10.3758/s13415-021-00883-z. PMC8490486 33821458

[R5] Becker MP, Nitsch AM, Miltner WH, & Straube T (2014). A single-trial estimation of the feedback-related negativity and its relation to BOLD responses in a time-estimation task. Journal of Neuroscience, 34(8), 3005–3012. 24553940 10.1523/JNEUROSCI.3684-13.2014 PMC6608516

[R6] Belden AC, Irvin K, Hajcak G, Kappenman ES, Kelly D, Karlow S, … Barch DM (2016). Neural correlates of reward processing in depressed and healthy preschool-age children. Journal of the American Academy of Child & Adolescent Psychiatry, 55(12), 1081–1089. 27871643 10.1016/j.jaac.2016.09.503 PMC5131532

[R7] Bouma EMC, Ormel J, Verhulst FC, & Oldehinkel AJ (2008). Stressful life events and depressive problems in early adolescent boys and girls: The influence of parental depression, temperament and family environment. Journal of Affective Disorders, 105(1–3), 185–193. 10.1016/j.jad.2007.05.007. 17574686

[R8] Bowyer CB, Joyner KJ, Yancey JR, Venables NC, Hajcak G, & Patrick CJ (2019). Toward a neurobehavioral trait conceptualization of depression proneness. Psychophysiology, 56(7), e13367. 10.1111/psyp.13367. 30950526

[R9] Bress JN, Foti D, Kotov R, Klein DN, & Hajcak G (2013). Blunted neural response to rewards prospectively predicts depression in adolescent girls. Psychophysiology, 50(1), 74–81. 23252717 10.1111/j.1469-8986.2012.01485.x

[R10] Bress JN, & Hajcak G (2013). Self-report and behavioral measures of reward sensitivity predict the feedback negativity. Psychophysiology, 50(7), 610–616. 10.1111/psyp.12053. 23656631

[R11] Bress JN, Smith E, Foti D, Klein DN, & Hajcak G (2012). Neural response to reward and depressive symptoms in late childhood to early adolescence. Biological Psychology, 89(1), 156–162. 22015709 10.1016/j.biopsycho.2011.10.004 PMC3245787

[R12] Bromet E, Andrade LH, Hwang I, Sampson NA, Alonso J, de Girolamo G, … Kessler RC (2011). Cross-national epidemiology of DSM-IV major depressive episode. BMC Medicine, 9(1), 90. 10.1186/1741-7015-9-90. 21791035 PMC3163615

[R13] Brush CJ, Ehmann PJ, Hajcak G, Selby EA, & Alderman BL (2018). Using multilevel modeling to examine blunted neural responses to reward in major depression. Biological Psychiatry: Cognitive Neuroscience and Neuroimaging, 3(12), 1032–1039. 29759821 10.1016/j.bpsc.2018.04.003

[R14] Bryce CA, & Floresco SB (2016). Perturbations in effort-related decision-making driven by acute stress and corticotropin-releasing factor. Neuropsychopharmacology, 41(8), 2147–2159. 10.1038/npp.2016.15. 26830960 PMC4908645

[R15] Burani K, Klawohn J, Levinson AR, Klein DN, Nelson BD, & Hajcak G (2021). Neural response to rewards, stress and sleep interact to prospectively predict depressive symptoms in adolescent girls. Journal of Clinical Child & Adolescent Psychology, 50(1), 131–140. 10.1080/15374416.2019.1630834. 31328972 PMC6980457

[R16] Byrne ML, Lind MN, Horn SR, Mills KL, Nelson BW, Barnes ML, … Allen NB (2021). Using mobile sensing data to assess stress: Associations with perceived and lifetime stress, mental health, sleep, and inflammation. Digital Health, 7, 20552076211037228. 10.1177/20552076211037227. PMC8580497 34777852

[R17] Carlson JM, Foti D, Mujica-Parodi LR, Harmon-Jones E, & Hajcak G (2011). Ventral striatal and medial prefrontal BOLD activation is correlated with reward-related electrocortical activity: A combined ERP and fMRI study. Neuroimage, 57(4), 1608–1616. 21624476 10.1016/j.neuroimage.2011.05.037

[R18] Carter JS, & Garber J (2011). Predictors of the first onset of a major depressive episode and changes in depressive symptoms across adolescence: Stress and negative cognitions. Journal of Abnormal Psychology, 120(4), 779–796. 10.1037/a0025441. 21928863

[R19] Casement MD, Shaw DS, Sitnick SL, Musselman SC, & Forbes EE (2015). Life stress in adolescence predicts early adult reward-related brain function and alcohol dependence. Social Cognitive and Affective Neuroscience, 10(3), 416–423. 10.1093/scan/nsu061. 24795442 PMC4350480

[R20] Cazassa MJ, da Oliveira MS, Spahr CM, Shields GS, & Slavich GM (2020). The stress and adversity inventory for adults (adult STRAIN) in Brazilian Portuguese: Initial validation and links with executive function, sleep, and mental and physical health. Frontiers in Psychology, 10, 3083. 10.3389/fpsyg.2019.03083. 32063871 PMC6999460

[R21] Felger JC, Li Z, Haroon E, Woolwine BJ, Jung MY, Hu X, & Miller AH (2016). Inflammation is associated with decreased functional connectivity within corticostriatal reward circuitry in depression. Molecular Psychiatry, 21(10), 1358–1365. 10.1038/mp.2015.168. 26552591 PMC4862934

[R22] Fletcher TD (2010). psychometric: Applied psychometric theory (version R package version 2.2). Retrieved from https://CRAN.R-project.org/package=psychometric.

[R23] Foti D, & Hajcak G (2009). Depression and reduced sensitivity to non-rewards versus rewards: Evidence from event-related potentials. Biological Psychology, 81(1), 1–8. 19162124 10.1016/j.biopsycho.2008.12.004

[R24] Fox J (2020). Companion to applied regression (version 3.0–10). Retrieved from https://r-forge.r-project.org/projects/car/, https://CRAN.R-project.org/package=car.

[R25] Fried EI, Nesse RM, Guille C, & Sen S (2015). The differential influence of life stress on individual symptoms of depression. Acta Psychiatrica Scandinavica, 131(6), 465–471. 10.1111/acps.12395. 25650176 PMC4428974

[R26] Furman D, Campisi J, Verdin E, Carrera-Bastos P, Targ S, Franceschi C, … Slavich GM (2019). Chronic inflammation in the etiology of disease across the life span. Nature Medicine, 25(12), 1822–1832. 10.1038/s41591-019-0675-0. PMC7147972 31806905

[R27] Ge X, Lorenz FO, Conger RD, Elder GH, & Simons RL (1994). Trajectories of stressful life events and depressive symptoms during adolescence. Developmental Psychology, 30(4), 467. 10.1037/0012-1649.30.4.467.

[R28] Goldstein BL, Kessel EM, Kujawa A, Finsaas MC, Davila J, Hajcak G, & Klein DN (2020). Stressful life events moderate the effect of neural reward responsiveness in childhood on depressive symptoms in adolescence. Psychological Medicine, 50(9), 1548–1555. 31274066 10.1017/S0033291719001557 PMC8101023

[R29] Gratton G, Coles MG, & Donchin E (1983). A new method for off-line removal of ocular artifact. Electroencephalography and Clinical Neurophysiology, 55(4), 468–484. 6187540 10.1016/0013-4694(83)90135-9

[R30] Greenberg PE, Fournier A-A, Sisitsky T, Pike CT, & Kessler RC (2015). The economic burden of adults with major depressive disorder in the United States (2005 and 2010). The Journal of Clinical Psychiatry, 76(02), 155–162. 10.4088/JCP.14m09298. 25742202

[R31] Hammen C (2005). Stress and depression. Annual Review of Clinical Psychology, 1(1), 293–319. 10.1146/annurev.clinpsy.1.102803.143938. 17716090

[R32] Hammen C, Kim EY, Eberhart NK, & Brennan PA (2009). Chronic and acute stress and the prediction of major depression in women. Depression and Anxiety, 26(8), 718–723. 10.1002/da.20571. 19496077 PMC3380803

[R33] Hanson JL, Albert D, Iselin A-MR, Carré JM, Dodge KA, & Hariri AR (2016). Cumulative stress in childhood is associated with blunted reward-related brain activity in adulthood. Social Cognitive and Affective Neuroscience, 11(3), 405–412. 10.1093/scan/nsv124. 26443679 PMC4769626

[R34] Harkness KL, & Monroe SM (2016). The assessment and measurement of adult life stress: Basic premises, operational principles, and design requirements. Journal of Abnormal Psychology, 125(5), 727. 10.1037/abn0000178. 27254487

[R35] Johnson PO, & Neyman J (1936). Tests of certain linear hypotheses and their application to some educational problems. Statistical Research Memoirs, 1, 57–93.

[R36] Kendler KS, Karkowski LM, & Prescott CA (1999). Causal relationship between stressful life events and the onset of major depression. American Journal of Psychiatry, 156(6), 837–841. 10.1176/ajp.156.6.837. 10360120

[R37] Kessler RC (1997). The effects of stressful life events on depression. Annual Review of Psychology, 48(1), 191–214. 10.1146/annurev.psych.48.1.191. 9046559

[R38] Kessler RC, Heeringa S, Lakoma MD, Petukhova M, Rupp AE, Schoenbaum M, … Zaslavsky AM (2008). Individual and societal effects of mental disorders on earnings in the United States: Results from the national comorbidity survey replication. American Journal of Psychiatry, 165(6), 703–711. 10.1176/appi.ajp.2008.08010126. 18463104 PMC2410028

[R39] Klawohn J, Burani K, Bruchnak A, Santopetro N, & Hajcak G (2020). Reduced neural response to reward and pleasant pictures independently relate to depression. Psychological Medicine, 51(5), 741–749. 31907094 10.1017/S0033291719003659

[R40] Klein DN, Shankman SA, Lewinsohn PM, & Seeley JR (2009). Subthreshold depressive disorder in adolescents: Predictors of escalation to full-syndrome depressive disorders. Journal of the American Academy of Child & Adolescent Psychiatry, 48(7), 703–710. 10.1097/CHI.0b013e3181a56606. 19465876 PMC2866498

[R41] Kovacs M (1992). Children’s depression inventory. New York: Multi-Health Systems North Tonawanda.

[R42] Kumar P, Berghorst LH, Nickerson LD, Dutra SJ, Goer FK, Greve DN, & Pizzagalli DA (2014). Differential effects of acute stress on anticipatory and consummatory phases of reward processing. Neuroscience, 266, 1–12. 10.1016/j.neuroscience.2014.01.058. 24508744 PMC4026279

[R43] Kupferberg A, Bicks L, & Hasler G (2016). Social functioning in major depressive disorder. Neuroscience and Biobehavioral Reviews, 69, 313–332. 10.1016/j.neubiorev.2016.07.002. 27395342

[R44] Levinson AR, Speed BC, Infantolino ZP, & Hajcak G (2017). Reliability of the electrocortical response to gains and losses in the doors task. Psychophysiology, 54(4), 601–607. 28072462 10.1111/psyp.12813

[R45] Lincoln SH, Pisoni A, Bondy E, Kumar P, Singleton P, Hajcak G, … Auerbach RP (2019). Altered reward processing following an acute social stressor in adolescents. PLoS One, 14(1), e0209361. 10.1371/journal.pone.0209361. 30608940 PMC6319717

[R46] Liu W, Wang L, Shang H, Shen Y, Li Z, Cheung EFC, & Chan RCK (2014). The influence of anhedonia on feedback negativity in major depressive disorder. Neuropsychologia, 53, 213–220. 10.1016/j.neuropsychologia.2013.11.023. 24316199

[R47] Long JA (2019). Interactions: comprehensive, user-friendly toolkit for probing interactions. R package ver., 1.1.0.

[R48] Luking KR, Nelson BD, Infantolino ZP, Sauder CL, & Hajcak G (2017). Internal consistency of functional magnetic resonance imaging and electroencephalography measures of reward in late childhood and early adolescence. Biological Psychiatry: Cognitive Neuroscience and Neuroimaging, 2(3), 289–297. 29057369 10.1016/j.bpsc.2016.12.004 PMC5647886

[R49] Lupien SJ, McEwen BS, Gunnar MR, & Heim C (2009). Effects of stress throughout the lifespan on the brain, behaviour and cognition. Nature Reviews Neuroscience, 10(6), 434–445. 10.1038/nrn2639. 19401723

[R50] Mazure CM (1998). Life stressors as risk factors in depression. Clinical Psychology: Science and Practice, 5(3), 291–313. 10.1111/j.1468-2850.1998.tb00151.x.

[R51] Meyer A, Lerner MD, De Los Reyes A, Laird RD, & Hajcak G (2017). Considering ERP difference scores as individual difference measures: Issues with subtraction and alternative approaches. Psychophysiology, 54(1), 114–122. 10.1111/psyp.12664. 28000251

[R52] Monroe SM, & Harkness KL (2011). Recurrence in major depression: A conceptual analysis. Psychological Review, 118(4), 655. 10.1037/a0025190. 21895384

[R53] Monroe SM, & Simons AD (1991). Diathesis-stress theories in the context of life stress research: Implications for the depressive disorders. Psychological Bulletin, 110(3), 406. 1758917 10.1037/0033-2909.110.3.406

[R54] Monroe SM, Slavich GM, & Georgiades K (2009). The social environment and life stress in depression. In Gotlib IH, & Hammen CL (Eds.), Handbook of depression (2nd edn., pp. 340–360). The Guilford Press: New York, NY, USA.

[R55] Nelson BD, Perlman G, Klein DN, Kotov R, & Hajcak G (2016). Blunted neural response to rewards as a prospective predictor of the development of depression in adolescent girls. American Journal of Psychiatry, 173(12), 1223–1230. 27363510 10.1176/appi.ajp.2016.15121524

[R56] Novick AM, Levandowski ML, Laumann LE, Philip NS, Price LH, & Tyrka AR (2018). The effects of early life stress on reward processing. Journal of Psychiatric Research, 101, 80–103. 10.1016/j.jpsy-chires.2018.02.002. 29567510 PMC5889741

[R57] Nusslock R, Young CB, & Damme KSF (2014). Elevated reward-related neural activation as a unique biological marker of bipolar disorder: Assessment and treatment implications. Behaviour Research and Therapy, 62, 74–87. 10.1016/j.brat.2014.08.011. 25241675 PMC6727647

[R58] Pegg S, Ethridge P, Shields GS, Slavich GM, Weinberg A, & Kujawa A (2019). Blunted social reward responsiveness moderates the effect of lifetime social stress exposure on depressive symptoms. Frontiers in Behavioral Neuroscience, 13, 178. 10.3389/fnbeh.2019.00178. 31447659 PMC6692494

[R59] Pizzagalli DA (2014). Depression, stress, and anhedonia: Toward a synthesis and integrated model. Annual Review of Clinical Psychology, 10(1), 393–423. 10.1146/annurev-clinpsy-050212-185606. PMC3972338 24471371

[R60] Porcelli AJ, Lewis AH, & Delgado MR (2012). Acute stress influences neural circuits of reward processing. Frontiers in Neuroscience, 6, 157. 10.3389/fnins.2012.00157. 23125822 PMC3485541

[R61] Proudfit GH (2015). The reward positivity: From basic research on reward to a biomarker for depression. Psychophysiology, 52(4), 449–459. 25327938 10.1111/psyp.12370

[R62] R Core Team (2020). R: A language and environment for statistical computing. Vienna, Austria: R Foundation for Statistical Computing. Retrieved from https://www.R-project.org/.

[R63] Revelle W (2020). Psych: Procedures for psychological, psychometric, and personality research (Version R package version 2.0.7). Evanston, IL: Northwestern University. Retrieved from https://CRAN.R-project.org/package=psych.

[R64] Rice F, Riglin L, Lomax T, Souter E, Potter R, Smith DJ, … Thapar A (2019). Adolescent and adult differences in major depression symptom profiles. Journal of Affective Disorders, 243, 175–181. 10.1016/j.jad.2018.09.015. 30243197

[R65] Slavich GM (2016). Life stress and health: A review of conceptual issues and recent findings. Teaching of Psychology, 43(4), 346–355. 10.1177/0098628316662768. 27761055 PMC5066570

[R66] Slavich GM (2020). Social safety theory: A biologically based evolutionary perspective on life stress, health, and behavior. Annual Review of Clinical Psychology, 16(1), annurev-clinpsy-032816–045159. 10.1146/annurev-clinpsy-032816-045159. PMC7213777 32141764

[R67] Slavich GM (2022). Social safety theory: Understanding social stress, disease risk, resilience, and behavior during the COVID-19 pandemic and beyond. Current Opinion in Psychology, 45, 101299. 10.1016/j.copsyc.2022.101299. 35219156 PMC8769662

[R68] Slavich GM, & Irwin MR (2014). From stress to inflammation and major depressive disorder: A social signal transduction theory of depression. Psychological Bulletin, 140(3), 774. 10.1037/a0035302. 24417575 PMC4006295

[R69] Slavich GM, O’Donovan A, Epel ES, & Kemeny ME (2010). Black sheep get the blues: A psychobiological model of social rejection and depression. Neuroscience and Biobehavioral Reviews, 35(1), 39–45. 10.1016/j.neubiorev.2010.01.003. 20083138 PMC2926175

[R70] Slavich GM, & Shields GS (2018). Assessing lifetime stress exposure using the stress and adversity inventory for adults (adult STRAIN): An overview and initial validation. Psychosomatic Medicine, 80(1), 17–27. 10.1097/PSY.0000000000000534. 29016550 PMC5757659

[R71] Slavich GM, Stewart JG, Esposito EC, Shields GS, & Auerbach RP (2019). The stress and adversity inventory for adolescents (adolescent STRAIN): Associations with mental and physical health, risky behaviors, and psychiatric diagnoses in youth seeking treatment. Journal of Child Psychology and Psychiatry, 60(9), 998–1009. 10.1111/jcpp.13038. 30912589 PMC6692180

[R72] Solomon DA, Keller MB, Leon AC, Mueller TI, Lavori PW, Shea MT, … Endicott J (2000). Multiple recurrences of major depressive disorder. American Journal of Psychiatry, 157(2), 229–233. 10.1176/appi.ajp.157.2.229. 10671391

[R73] Sturmbauer SC, Shields GS, Hetzel E-L, Rohleder N, & Slavich GM (2019). The stress and adversity inventory for adults (adult STRAIN) in German: An overview and initial validation. PLoS One, 14(5), e0216419. 10.1371/journal.pone.0216419. 31071135 PMC6508721

[R74] Van Pett K, Viau V, Bittencourt JC, Chan RKW, Li H-Y, Arias C, … Sawchenko PE (2000). Distribution of mRNAs encoding CRF receptors in brain and pituitary of rat and mouse. Journal of Comparative Neurology, 428 (2), 191–212. 10.1002/1096-9861(20001211)428:2<191::AID-CNE1>3.0.CO;2-U. 11064361

[R75] van Praag HM (2004). Can stress cause depression? Progress in Neuro-Psychopharmacology and Biological Psychiatry, 28(5), 891–907. 10.1016/j.pnpbp.2004.05.031. 15363612

[R76] Wight RG, Aneshensel CS, Botticello AL, & Sepúlveda JE (2005). A multilevel analysis of ethnic variation in depressive symptoms among adolescents in the United States. Social Science & Medicine, 60(9), 2073–2084. 10.1016/j.socscimed.2004.08.065. 15743655

