# Supplementary material for: Cumulative lifetime acute stressor exposure interacts with reward responsiveness to predict longitudinal increases in depression severity in adolescence
Source: Psychol Med. Author manuscript; Available in PMC 2023 Sep 13. (PMC10388334; doi:10.1017/S0033291722001386)
Supplement: Supplement [file NIHMS1876184-supplement-Supplement.docx]

**Supplementary Materials for**

**Cumulative Lifetime Acute Stressor Exposure Interacts with Reward Responsiveness to Predict Longitudinal Increases in Depression Severity in Adolescence**

**Supplementary Table S1.**

*Regression results using depressive symptoms at follow up as the dependent variable predicted from lifetime acute stressor exposure, lifetime chronic stressor exposure, baseline age, RewP, depressive symptoms, household income and ethnicity, the lifetime acute stressor × RewP interaction, and the lifetime chronic stressor exposure × RewP interaction*.

| Predictor | *b* | *b*  95% CI  [LL, UL] | *VIF* | Model Fit |
| --- | --- | --- | --- | --- |
| (Intercept) | 6.13** | [3.16, 9.10] | - |  |
| Baseline RewP | -0.07 | [-0.17, 0.04] | 1.06 |  |
| Baseline Age | 0.33 | [-0.05, 0.72] | 1.17 |  |
| Baseline Depressive Symptoms (CDI) | 0.34** | [0.22, 0.46] | 1.40 |  |
| Lifetime Chronic Stressor Exposure (STRAIN) | 0.42** | [0.22, 0.61] | 2.38 |  |
| Lifetime Acute Stressor Exposure (STRAIN) | 0.08 | [-0.06, 0.22] | 2.20 |  |
| Ethnicity | 0.15 | [-1.79, 2.09] | 1.08 |  |
| Household Income | 0.31 | [-2.70, 3.31] | 1.02 |  |
| Lifetime Acute Stressor Exposure*Baseline RewP | -0.01^ | [-0.04, 0.01] | 2.07 |  |
| Lifetime Chronic Stressor Exposure*Baseline RewP | -0.00 | [-0.04, 0.03] | 2.07 |  |
|  |  |  |  | *R^2^*  = .474** |
|  |  |  |  | 95% CI [.35, .53] |

*Note.* RewP = Reward Positivity; CDI = Children’s Depression Inventory; STRAIN = Stress and Adversity Inventory; *b* = unstandardized regression weights; CI = 95% confidence interval; *LL* and *UL* indicate the lower and upper limits of the CI, respectively. ^ = *p* < .10; * = *p* < .05; ** = *p* < .01.

**Supplementary Table S2.**

*Regression results using depressive symptoms at follow up as the dependent variable predicted from lifetime acute stressor severity, lifetime chronic stressor severity, baseline age, RewP, depressive symptoms, the lifetime acute severity × RewP interaction, and the lifetime chronic severity exposure × RewP interaction.*

| Predictor | *b* | *b*  95% CI  [LL, UL] | *VIF* | Model Fit |
| --- | --- | --- | --- | --- |
| (Intercept) | 6.66** | [6.07, 7.24] |  |  |
| Baseline RewP | -0.07 | [-0.16, 0.03] | 1.07 |  |
| Baseline Age | 0.23 | [-0.12, 0.58] | 1.17 |  |
| Baseline Depressive Symptoms (CDI) | 0.37** | [0.26, 0.47] | 1.37 |  |
| Lifetime Chronic Stressor Severity (STRAIN) | 0.13** | [0.08, 0.19] | 2.70 |  |
| Lifetime Acute Stressor Severity (STRAIN) | 0.05 | [-0.04, 0.14] | 2.36 |  |
| Lifetime Acute Stressor Severity × Baseline RewP | -0.02* | [-0.03, -0.00] | 2.61 |  |
| Lifetime Chronic Stressor Severity × Baseline RewP | 0.00 | [-0.01, 0.01] | 2.67 |  |
|  |  |  |  | *R^2^*  = .513** |
|  |  |  |  | 95% CI [.41,.57] |

*Note.* RewP = Reward Positivity; CDI = Children’s Depression Inventory; STRAIN = Stress and Adversity Inventory; *b* = unstandardized regression weights; CI = 95% confidence interval; *LL* and *UL* indicate the lower and upper limits of the CI, respectively. * = *p* < .05. ** = *p* < .01.

**Supplementary Table S2a.**

*Simple slopes of the relation between lifetime acute stressor severity (STRAIN) and depression severity (CDI scores) two years later at different levels of baseline RewP.*

| *Lifetime Acute Stress Severity* × *Baseline* *RewP* |  |  |  |  |
| --- | --- | --- | --- | --- |
|  | *b* | Lower CI | Upper CI | *p* |
| RewP (-1 *SD*) | 0.16 | 0.02 | 0.30 | 0.027* |
| RewP (*M*) | 0.05 | - 0.04 | 0.14 | 0.284 |
| RewP (+1 *SD*) | - 0.10 | - 0.20 | 0.07 | 0.334 |

*Note:* RewP = Reward Positivity; CI = 95% confidence interval. * = *p* < .05. Beta coefficients are

adjusted for baseline depressive symptoms and baseline age.

**Supplementary Table S3.**

*Regression results using depressive symptoms at follow up as the dependent variable predicted from lifetime acute stressor exposure, lifetime chronic stressor exposure, baseline age, baseline depressive symptoms, and baseline residualized wins, the lifetime acute stressor exposure × baseline residualized wins interaction, and the lifetime chronic stressor exposure × baseline residualized wins interaction.*

| Predictor | *b* | *b*  95% CI  [LL, UL] | *VIF* | Model Fit |
| --- | --- | --- | --- | --- |
| (Intercept) | 6.60** | [6.01, 7.20] | - |  |
| Baseline Residualized Wins | -0.07 | [-0.16, 0.03] | 1.10 |  |
| Baseline Age | 0.34 | [-0.03, 0.70] | 1.22 |  |
| Baseline Depressive Symptoms (CDI) | 0.38** | [0.28, 0.49] | 1.36 |  |
| Lifetime Chronic Stressor Exposure (STRAIN) | 0.44** | [0.26, 0.63] | 2.34 |  |
| Lifetime Acute Stressor Exposure (STRAIN) | 0.07 | [-0.07, 0.20] | 2.17 |  |
| Lifetime Acute Stressor Exposure × Baseline Residualized Wins | -0.02* | [-0.05, -0.00] | 2.09 |  |
| Lifetime Chronic Stressor Exposure × Baseline Residualized Wins | 0.01 | [-0.02, 0.04] | 2.08 |  |
|  |  |  |  | *R^2^*  = .497** |
|  |  |  |  | 95% CI [.40,.56] |

*Note.* RewP = Reward Positivity; CDI = Children’s Depression Inventory; STRAIN = Stress and Adversity Inventory; *b* = unstandardized regression weights; CI = 95% confidence interval; *LL* and *UL* indicate the lower and upper limits of the CI, respectively. * = *p* < .05; ** = *p* < .01.

**Supplementary Table S3a.**

*Simple slopes of the relation between lifetime acute stressor exposure (STRAIN) and depression severity (CDI scores) two years later at different levels of residualized wins.*

| *Lifetime Acute Stressor Exposure* × Residualized Wins |  |  |  |  |
| --- | --- | --- | --- | --- |
|  | *b* | Lower CI | Upper CI | *p* |
| Baseline Residualized Wins (-1 *SD*) | 0.23 | 0.03 | 0.42 | 0.024* |
| Baseline Residualized Wins (*M*) | 0.07 | - 0.07 | 0.20 | 0.316 |
| Baseline Residualized Wins (+1 *SD*) | - 0.09 | - 0.29 | 0.11 | 0.364 |

*Note:* RewP = Reward Positivity; CI = 95 % confidence intervals. **p* < .05. Beta coefficients are

adjusted for baseline depressive symptoms and baseline age.

**Supplementary Table S4.**

*Regression results using depressive symptoms at follow up as the dependent variable predicted from lifetime acute stressor exposure, lifetime chronic stressor exposure, baseline age, baseline depressive symptoms, baseline residualized losses, the lifetime acute stressor exposure × baseline residualized losses interaction, and the lifetime chronic stressor exposure × baseline residualized losses interaction.*

| Predictor | *b* | *b*  95% CI  [LL, UL] | *VIF* | Model Fit |
| --- | --- | --- | --- | --- |
| (Intercept) | 6.63** | [6.04, 7.23] | - |  |
| Baseline Residualized Losses | 0.01 | [-0.09, 0.11] | 1.02 |  |
| Baseline Age | 0.30 | [-0.06, 0.65] | 1.14 |  |
| Baseline Depressive Symptoms (CDI) | 0.37** | [0.27, 0.48] | 1.36 |  |
| Lifetime Chronic Stressor Exposure (STRAIN) | 0.44** | [0.25, 0.63] | 2.36 |  |
| Lifetime Acute Stressor Exposure (STRAIN) | 0.08 | [-0.06, 0.21] | 2.16 |  |
| Lifetime Acute Stressor Exposure × Baseline Residualized Losses | 0.02 | [-0.01, 0.04] | 2.34 |  |
| Lifetime Chronic Stressor Exposure × Baseline Residualized Losses | -0.00 | [-0.03, 0.02] | 2.35 |  |
|  |  |  |  | *R^2^*  = .487** |
|  |  |  |  | 95% CI [.39, .55] |

*Note.* RewP = Reward Positivity; CDI = Children’s Depression Inventory; STRAIN = Stress and Adversity Inventory*; b* = unstandardized regression weights; CI = 95% confidence interval; *LL* and *UL* indicate the lower and upper limits of the CI, respectively. ** = *p* < .01.
